# Imaging in Myotonic Dystrophy Type 1 – Case Reports

**DOI:** 10.5334/jbr-btr.994

**Published:** 2016-10-10

**Authors:** Jurgen Bielen, Steven Schepers, Bruno Termote, Rik Vanwyck, Geert Souverijns

**Affiliations:** 1 UZ Leuven, BE; 2 Jessa Ziekenhuis, BE

**Keywords:** Myotonic dystrophy type 1, Temporal, White matter T2 hyperintensity

## Abstract

Myotonic dystrophy type 1 (DM1) is the most common of the muscular dystrophies. It is an autosomal dominant neuromuscular disorder with multisystem involvement, including the central nervous system. Two DNA-proven cases are presented. Patients reported are siblings showing features of DM1 on magnetic resonance imaging (MRI). These features include T2 and FLAIR hyperintensities in the periventricular, deep, and subcortical white matter, with frequent involvement of the anterior temporal lobe. Other features include general brain atrophy and enlarged Virchow-Robin spaces. Subcortical white matter lesions anterior in the temporal lobe are the most specific imaging finding, and a short differential diagnosis is discussed.

## Introduction

With a prevalence of 1 in 8,000, myotonic dystrophy (DM) is the most common form of adult-onset muscular dystrophy. It is an autosomal dominant inherited, multisystemic disorder with two major forms: DM1, also known as Steinert’s disease, and DM2, a milder version. The adult-onset form of DM1 typically presents at 15 to 40 years. It mainly affects the neuromuscular system, but it can also affect the eye, heart, endocrine system, and central nervous system [1]. Clinical presentation and familial history form the basis of the diagnostic process, with specific genetic testing being the gold standard for confirming the diagnosis. Other useful diagnostic studies include electromyography and muscle biopsy [1]. Brain magnetic resonance imaging can also show features compatible with DM1 [2]. Herein, we present these findings in two cases of adult-onset DM1 of siblings of nonconsanguineous parents.

## Case Reports

Patient A, a 43-year-old woman, presented to the neurologist with increased difficulty swallowing and speaking, stiffness in the jaw muscles, and weight loss. Previous history reveals partial thyroidectomy (unknown cause), hypothyroidism, and an episode of atrial fibrillation due to overcompensated hypothyroidism. On clinical examination, there was a myotonic handshake, myopathic facial expression, and a positive Gower’s sign (weakness of the proximal muscles). Electromyography showed frequent myotonic discharges. Electrocardiography was normal. A barium study of the hypopharynx showed decreased motility of the hypopharynx and a deficient closing of the upper gastrointestinal sphincter.

**Figure 1:**
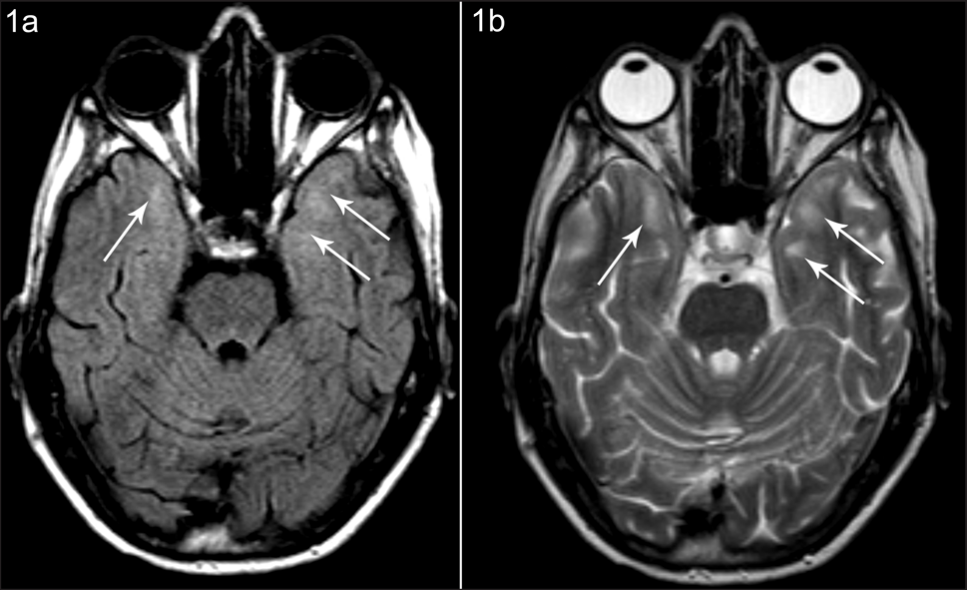
Patient A, axial FLAIR **(a)** and T2-weighted **(b)** images at the same level show subcortical white matter hyperintensity anteromedial in the temporal lobes, more pronounced on the left side.

**Figure 2:**
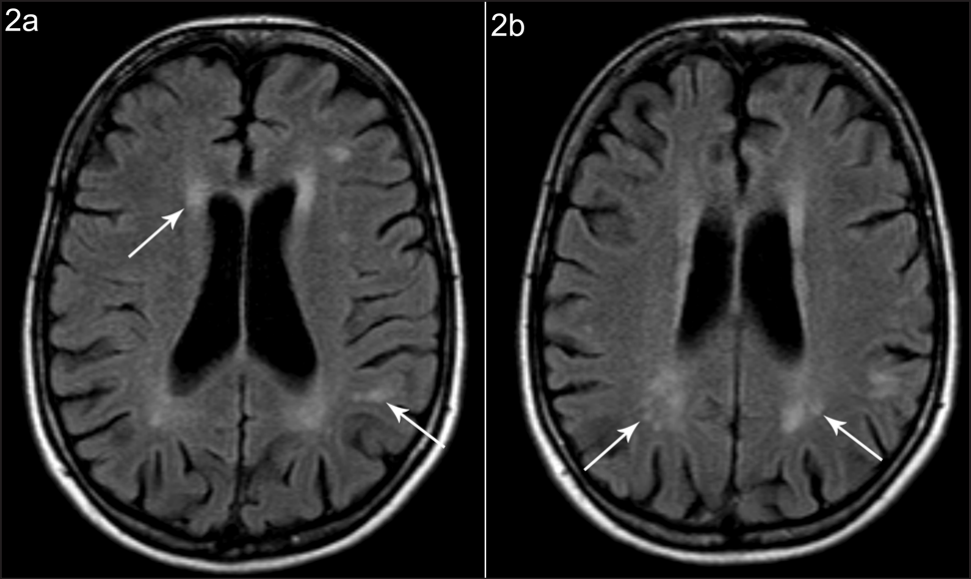
Patient A, axial FLAIR weighted images at different sections show **(a)** periventricular white matter hyperintensity and multiple hyperintense lesions in the subcortical white matter, with partial confluence and **(b)** white matter hyperintensity posterior and superior to the trigone.

**Figure 3:**
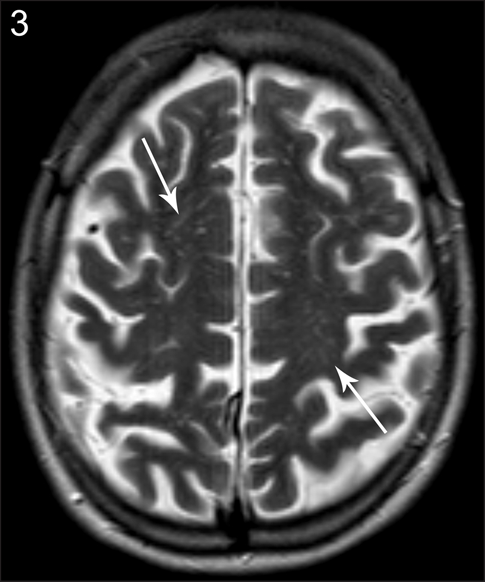
Patient A, axial T2-weighted image shows prominent Virchow-Robin spaces bilateral in the centrum semiovale.

An MRI of the brain showed multifocal T2 and FLAIR hyperintensities, with beginning confluence (Fazekas grade 1–2) located in the periventricular white matter and in the subcortical white matter of the frontal, parietal, and temporal lobes (anteromedial). There were also hyperintense lesions in the white matter posterior and superior to the trigone (Figures 1 and 2). Enlarged Virchow-Robin spaces were found in the centrum semiovale and less pronounced in the basal ganglia (Figure 3). Brainstem, cerebellar hemispheres, and gray matter were unremarkable. Diffusion-weighted and contrast-enhanced imaging were normal.

**Figure 4:**
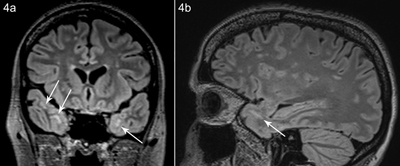
Patient B, coronal **(a)** and sagittal **(b)** FLAIR image shows white matter hyperintensity subcortical anteromedial in the temporal lobe.

Patient B, 42 years old and a sister of patient A, presented to the neurologist for evaluation of possible myotonic dystrophy. Clinical examination revealed a slight myotic handshake, myopathic facial expression, and a bilateral Bell’s sign (suggestive of weakness of the orbicularis orbis muscles). Cardiac evaluation was normal. An MRI of the brain showed similar findings as patient A, although less pronounced (Figure 4).

Genetic testing was performed in both patients, which showed an expansion of 800 CTG-repeats in patient A and an expansion of 600 CTG-repeats in patient B, confirming the diagnosis of myotonic dystrophy in both.

## Discussion

Myotonic dystrophy is a chronic, slowly progressing, and highly variable multisystemic disorder, with autosomal dominant inheritance. The characterizing neuromuscular features of classical adult-onset DM1 (ADM1) are skeletal muscle weakness, myotonia, and muscle pain. The muscle weakness occurs most frequently in the face, distal forearm, hand, and ankle dorsiflexors. Myotonia is best appreciated in the fingers but becomes less apparent when muscle weakness progresses over time. Neuromuscular symptoms can be accompanied (or even dominated) by cataracts, cardiac conduction abnormalities, infertility, insulin resistance, and irritable bowel symptoms. Three phenotypes of DM1 exist: congenital, mild adult-onset, and classical adult-onset [3]. While mental retardation is common in the congenital form of DM1, there are usually only mild to moderate cognitive and personality defects in the adult forms [1].

In ADM1, common findings are T2 and FLAIR hyperintensities in the periventricular and deep white matter, as well as in the subcortical white matter, with frequent involvement of the anterior temporal lobe [2–4]. General brain atrophy and dilated Virchow-Robin spaces are also commonly described, with dilated large convexity Virchow-Robin spaces more prominent in earlier disease states [5]. In the congenital form, there is more severe cerebral atrophy, ventriculomegaly, and hyperintensities in the white matter of the posterior and superior trigone [6].

Studies have shown that the MRI findings in ADM1 are related to the duration of the disease, and patients with short disease duration can have normal imaging findings. This is compatible with a degenerative origin of the white matter lesions (slow demyelination) [2]. Although the cortical and deep gray matter appears normal on conventional MRI, studies with voxel-based morphometry have shown a decrease in gray matter in DM1, most notably in the frontal and parietal regions and in the basal ganglia and thalami. Diffusor tension imaging has shown that the white matter is more affected than the gray matter and that the white matter involvement is much larger than the lesions found on T2-weighted MRIs [7].

The most common MR finding is T2 white matter hyperintensities in the anterior temporal lobes, which can also be seen in cerebral autosomal dominant arteriopathy with subcortical infarcts and leukoencephalopathy (CADASIL), cytomegalovirus (CMV) infection, and megalencephalic leukoencephalopathy [3]. Differentiating features of CADASIL are clinical presentation with recurrent strokes or migraine, lacunar infarcts, and lesions with restricted diffusion [8]. In CMV infection, the white matter lesions are usually most prominent in the parietal regions, combined with periventricular calcifications and cystic changes. Megalencephalic leukoencephalopathy shows cystic changes in the frontal and temporal subcortical regions, along with macrocephaly [3].

Although the MR findings are nonspecific, they can be helpful to the referring clinician as additional arguments for DM1. The gold standard for diagnosis remains specific genetic testing. DM1 results from an expansion of a CTG-trinucleotide repeat in dystrophia myotonica protein kinase gene (DMPK gene) on chromosome 19q 13.3. Affected individuals have repeats in the hundreds to thousands, compared to less than 34 CTG-repeats in normal individuals [1]. The size of the CTG-repeat expansion can be used as a biomarker for disease severity, and although still controversial, larger CTG-repeat expansions seem to be associated with more severe imaging findings [7].
